# SAR Image Simulation in the Time Domain for Moving Ocean Surfaces

**DOI:** 10.3390/s130404450

**Published:** 2013-04-02

**Authors:** Takero Yoshida, Chang-Kyu Rheem

**Affiliations:** 1 Department of Ocean Technology, Policy and Environment, The University of Tokyo, 4-6-1 Komaba, Meguro-ku, Tokyo 153-8505, Japan; 2 Institute of Industrial Science, The University of Tokyo, 4-6-1 Komaba, Meguro-ku, Tokyo 153-8505, Japan; E-Mail: rheem@iis.u-tokyo.ac.jp

**Keywords:** SAR, time domain simulation, ocean, remote sensing

## Abstract

This paper presents a fundamental simulation method to generate synthetic aperture radar (SAR) images for moving ocean surfaces. We have designed the simulation based on motion induced modulations and Bragg scattering, which are important features of ocean SAR images. The time domain simulation is able to obtain time series of microwave backscattering modulated by the orbital motions of ocean waves. Physical optics approximation is applied to calculate microwave backscattering. The computational grids are smaller than transmit microwave to demonstrate accurate interaction between electromagnetic waves and ocean surface waves. In this paper, as foundations for SAR image simulation of moving ocean surfaces, the simulation is carried out for some targets and ocean waves. The SAR images of stationary and moving targets are simulated to confirm SAR signal processing and motion induced modulation. Furthermore, the azimuth signals from the regular wave traveling to the azimuth direction also show the azimuthal shifts due to the orbital motions. In addition, incident angle dependence is simulated for irregular wind waves to compare with Bragg scattering theory. The simulation results are in good agreement with the theory. These results show that the simulation is applicable for generating numerical SAR images of moving ocean surfaces.

## 1. Introduction

Sea surface observations with a SAR from satellites or airplanes are effective methods to obtain global ocean data. Simulation technique of SAR images can contribute to verifying analytical algorithms of sea surface observations by SAR images. If conventional algorithms are applied to the simulated SAR images, it is possible to evaluate the accuracy of the estimated sea surface data by comparing them to the sea surface profiles, which are the initial parameters in the simulation. The relation between ocean conditions and SAR images is clear in the simulation. Thus the simulation is helpful to consider translation of wave features in the SAR images. Moreover, the simulation provides us fundamental understanding on imaging mechanisms in ocean area. The simulation is expected to attain more insight of ocean SAR images.

It is known that SAR images in oceanic scenes include velocity bunching caused by the orbital motions of ocean waves [1–3]. The velocity bunching leads to a nonlinear imaging mechanism in ocean SAR images. Accordingly, ocean waves shown in the SAR images have a “wavelike pattern”. The wavelike patterns in the SAR images are not linked to the ocean waves under the conditions of nonlinear imaging by velocity bunching. Therefore, it is difficult to analyze ocean data from the SAR images.

In order to simulate the ocean SAR images including velocity bunching, we need to consider the time series of microwave backscattering with motion effects. Thus, time domain is concerned in the simulation. In addition to velocity bunching, the SAR image simulation of ocean areas should be based on Bragg scattering [4], which is the primary mechanism of microwave backscattering on the sea surface.

So far, we have already developed a time domain simulation of microwave backscattering from numerical sea surface data with a fixed radar [5]. To calculate the microwave backscattering, physical optics (PO) approximation is applied to the simulation [6]. PO can obtain electric scattering fields by surface integral of surface electric currents induced by microwaves. Phases of the backscattered signals from each computational grid, which are smaller than the microwave, are computed by the physical optics approximation. Consequently, PO is able to calculate microwave backscattering based on Bragg scattering, that is a major component of microwave backscattering from the ocean surface. This simulation technique has been improved for the SAR image simulation in time domain of ocean surface composed of long waves and wind waves. The simulation is developed as a foundation to understand SAR imaging mechanism of moving ocean surface and to evaluate algorithms of sea surface observations using SAR images.

Other SAR image simulators are available for sea surface observations. There are velocity bunching (VB) [2,7,8] model and distributed surface (DS) model [9–11] for SAR image simulation of ocean areas. The simulators of VB models are based on modulation transfer function (MTF), which is addressed by Hasselmann *et al.* [12]. MTF is composed of tilt, hydrodynamic and velocity bunching modulations. According to Nunziata *et al.* [8], VB theory focuses on simple particle scattering. On the other hand, DS model focuses backscattering intensity from the surface. SAR image intensity of each facet is computed to generate numerical SAR images. However, DS model is not aimed to velocity bunching.

SARAS [10], which is based on DS model, is developed in time domain to obtain SAR raw signals. They use PO to compute backscattering intensity of each computational grid. The scale of the computational grids is one-quarter of the resolution cell. SAR signals from the grids are calculated in this simulator.

The beneficial features and some limitations of our simulator are described as follows. The simulation process of obtaining microwave backscattering is similar to that of an actual SAR system. In our simulator, the pulse irradiation area is the calculation area. It is divided into computational grids that are smaller than the wavelength of the transmitted microwave to demonstrate accurate interaction between electromagnetic waves and ocean surface waves. PO is also used to calculate microwave backscattering. The time series of microwave backscattering as SAR raw signals is the summation of backscattered microwave from all computational grids in the pulse irradiation area. The position of the calculation area changes with microwave pulse and platform motions to obtain SAR raw signals for range and azimuth directions.

One advantage of our simulator is that the phases of the received signals are based on Bragg scattering. The backscattered microwaves from computational grids smaller than the microwave are emphasized in the Bragg resonant condition. Therefore the simulation adequately reproduces microwave backscattering from the ocean surface. In addition, our simulator can also obtain the time series of SAR raw signals while considering modulations caused by moving ocean waves. In order to understand sophisticated SAR imaging mechanism of ocean waves, we attempt to develop a simulator that generates numerical SAR images with regard to not only motion induced modulations but also scattering intensity based on Bragg scattering. Note that our simulation is not suitable for land SAR images because it ignores shadowing and multi scattering, which are important imaging factors for SAR images of land areas. However, these are considered to be a minor scattering mechanism in ocean SAR images. In the ocean case, motion-induced modulation and microwave scattering on the sea surface are the key factors. Consequently, we focus on these factors in the SAR image simulation for moving ocean surfaces.

This paper is organized as follows: firstly, we have simulated a stationary target case to confirm SAR signal processing. The SAR raw signals of the range and the azimuth direction are simulated, and compression techniques are conducted to obtain SAR images with fine resolutions. The SAR images of moving targets are also simulated in the case of a moving target to investigate azimuthal shift [13,14], which is produced by the target's velocity. Next, the simulation is conducted in the case of the regular wave to confirm the azimuthal shift caused by the orbital motions. The displacements by the orbital motions become velocity bunching in the SAR images of long ocean waves. Moreover, SAR signals in the range direction are simulated for wind waves to confirm backscattering features on the sea surface. The incident angle dependence, which is the relation between SAR intensity and incident angles, is evaluated by comparing the theory of Bragg scattering. As the simulation results, we will show that the time domain simulation is applicable for generating numerical SAR images of moving ocean surfaces with regard to motion effects and Bragg scattering.

## 2. Simulation Method

### 2.1. Time Domain Simulation of SAR Signals by Physical Optics

**Figure 1.**
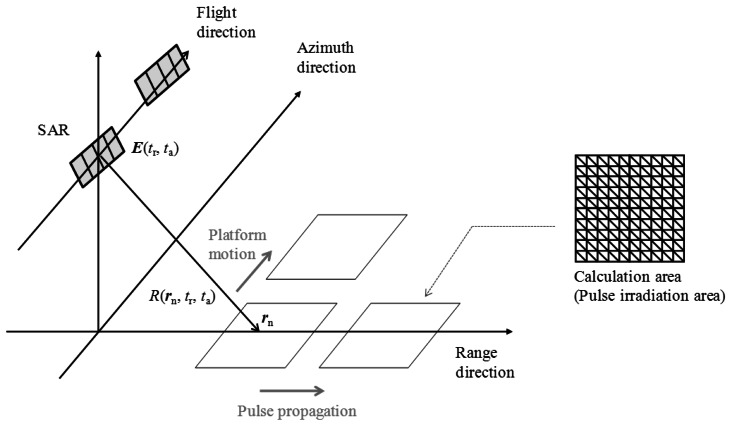
Conceptual figure of SAR image simulation in time domain.

Figure 1 illustrates the conceptual figure of the simulation. In this simulation, a physical optics approximation is applied to calculate the scattering electric field ***E*** by the surface integral of the induced surface electric currents ***J*** [6]. The calculation area is divided into the number of *N*. The position of the nth grid is ***r****_n_*, and the area of total computational grids is *A_n_*. The discrete surface integral of the scattering electric field ***E*** is expressed as follows:

(1) $$\text{E}(t_{\text{r}},t_{\text{a}}\;)\;=-i2\pi f_{c}\;\mu \sum_{n=1}^{N}\;\left(\frac{A_{\text{n}}}{4\pi R(r_{\text{n}},t_{\text{r}},t_{\text{a}})}J_{\text{n}}\left(r_{\text{n}},t_{\text{r}},t_{\text{a}}\right)\right)$$

(2) $$J\;(r_{\text{n}},t_{\text{r}},t_{\text{a}})=2n\times H\;(r_{\text{n}},t_{\text{r}},t_{\text{a}})$$

where *t_r_* and *t_a_* are the sampling timings in the range and the azimuth directions. *R* is the distance from the radar and the each computational grid, *μ* is the magnetic permeability, ***n*** is the surface normal vector of each computational grid, *f_c_* is the center frequency of the transmitted microwave, ***H*** is the incident magnetic field. It is assumed that the antenna is far from the sea surface.

In the SAR signal processing, the range and azimuth signals are based on chirp pulse Doppler shifts to obtain fine resolutions [15]. Thus, the magnetic field is expressed as following equation to calculate the SAR signals with regard to chirp pulse and Doppler shifts:

(3) $$H\;(r_{\text{n}},t_{\text{r}},t_{\text{a}})=\sqrt{\frac{Pɛ}{\mu }}\exp(i2\pi f_{c}(2R(r_{\text{n}},t_{\text{r}},t_{\text{a}})/c)+i\alpha \;{\left(2(R_{c}(t_{\text{r}},t_{\text{a}})-R(r_{\text{n}},t_{\text{r}},t_{\text{a}}))/c\right)}^{2})$$

where *ε* is the dielectric constant, and *P* is the transmit antenna beam power. *α* is the linear chirp rate, *R*_c_ is the distane from the radar and the center of the chirp pulse, *c* is the light velocity. Doppler shifts caused by movements of radar are included in the first term of the Equation (3) as changes of relative distances. Also, the frequency modulation of a chirp pulse is expressed in the second term of the Equation (3).

The advantage of PO is that electric scattering fields from each computational grid can be calculated independently. Therefore, PO is able to calculate microwave backscattering efficiently. It is reported that the computational grid is enough as 1/5 of microwave to calculate microwave backscattering accurately by PO in far fields [5].

The beam pattern of a planer antenna is described as sinc function. The antenna beam patterns with regard to vertical and horizontal planes *P_V_* (*β_V_*), *P_H_* (*β_H_*) are expressed as follows. *β_V_*, *β_H_* are the angles of vertical and horizontal planes between look angle and each element of the surface:

(4) $$P_{V}\left(\beta_{V}\right)={\left|\text{sinc}\;\left(\frac{\pi D_{V}}{\lambda_{e}}\sin(\beta_{V})\right)\right|}^{2}$$

(5) $$P_{H}\left(\beta_{H}\right)={\left|\text{sinc}\;\left(\frac{\pi D_{H}}{\lambda_{e}}\sin(\beta_{H})\right)\right|}^{2}$$

where *D_V_*, *D_H_* are the antenna lengths of vertical and horizontal planes. λ*_e_* is the wavelength of microwave. In the Equation (3), the antenna beam power *P* is composed of range and azimuth beam. It is rewritten as following equation:

(6) $$P=\frac{P_{V}P_{H}}{4\pi R^{2}}$$

**Figure 2.**
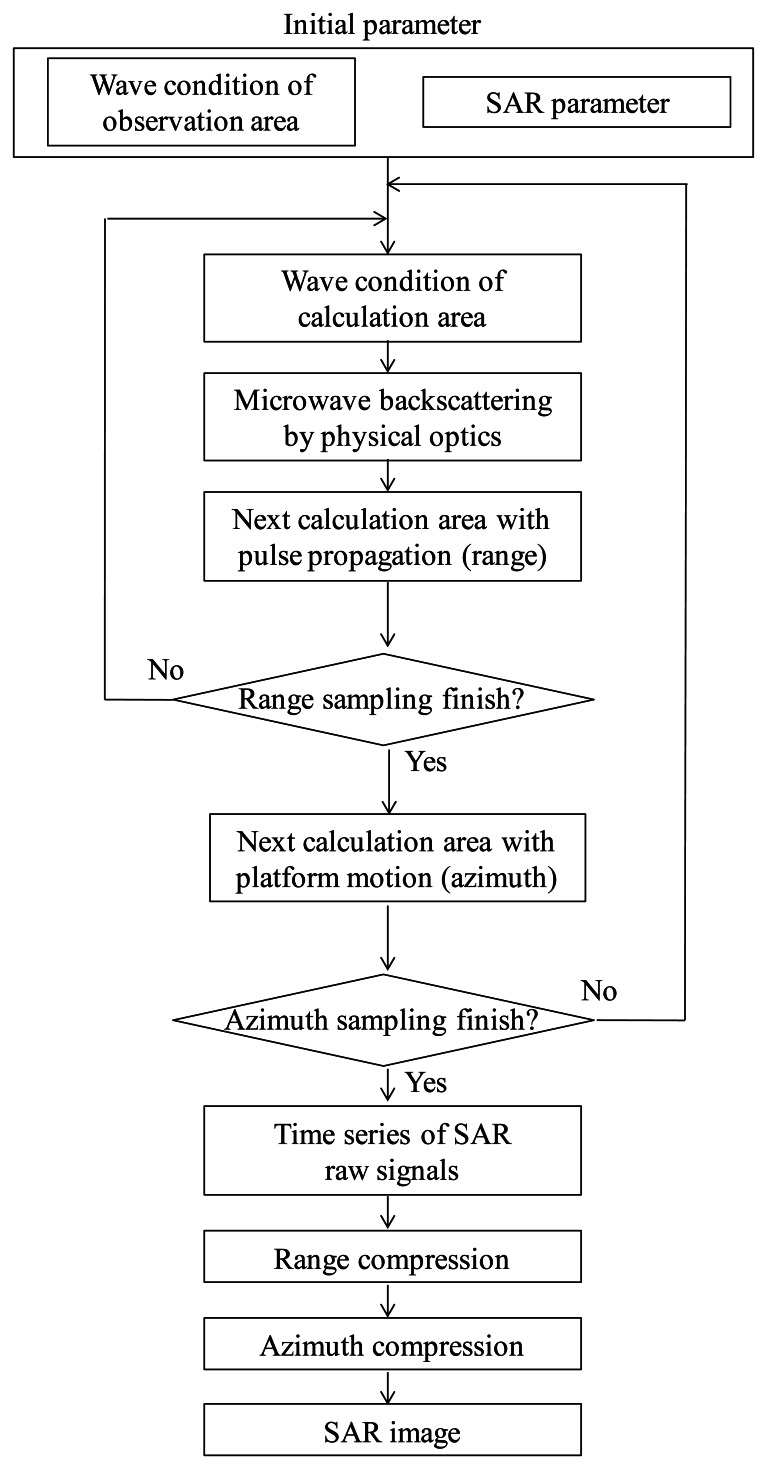
Flowchart of the simulation.

The flowchart of the simulation is shown in Figure 2. At first, the surface shape in observation areas and the parameters in terms of SAR are determined as initial parameters of the simulation. Next, microwave backscattering from the calculation area (microwave pulse irradiation area) is computed.

As illustrated in Figure 1, the calculation area is the microwave radiation area. The time series of the received signals are obtained as the summations of the scattering electric fields of all computational grids. The calculation area moves with sampling time of range and azimuth directions, respectively. Microwave backscattering from computational grids in the pulse irradiation area is calculated at each sampling time. In the range direction, the received signals are calculated with the movements of the radar pulse. The calculation area is changed with regard to pulse propagation at the intervals of range sampling. After sampling in one line of the range direction, the calculation area is moved to the azimuth direction. The calculation area is changed with regard to platform motion at the intervals of azimuth sampling. Continuously, the range signals are sampled with varying azimuth directions. The calculation area is altered within the observation area of range and azimuth directions. Then, the time series of SAR signals are obtained. These simulation steps are similar to that of actual SAR systems to obtain the time series of SAR raw signals. Finally, SAR images with fine resolutions are obtained by applying range and azimuth compression using FFT. As compression techniques, the convolutions are performed in the frequency domain between the raw data and the reference signal ***E****_ref_*, which is a complex conjugation signal from a point target. The compressed signal ***E****_c_* is shown in the following equation, where asterisk denotes convolution. ***E****_ref_* includes a Gaussian window function:

(7) $$E_{c}=E\;\left(t_{\text{r}},t_{\text{a}}\right)*E_{ref}$$

### 2.2. Simulation Condition

The simulation conditions are listed in Table 1. We assume the simulation scale as an airborne SAR. In the simulation, the wavelength of the microwave is 0.235 m. The computational grid size is 0.047 m, *i.e.*, 1/5 of the radar wavelength. The calculation area (pulse irradiation area on the sea surface) is 100 m for the range direction and 80 m for the azimuth direction. The velocity of the platform is 75 m/s, the altitude of the SAR is 1,500 m, and the look angle of the SAR is 40 degrees. The resolution of the azimuth direction depends on the azimuth antenna length, and the resolution is approximately 3 m. The calculation areas contain the antenna main lobe of the azimuth direction. The range resolution is determined by the chirp rate and the transmit pulse. The range resolution calculated by the chirp and the transmit pulse is approximately 4.5 m. The polarization in this simulation is HH, thus the horizontal components of microwave scattering are calculated in the simulation. The simulation area of this paper is relatively small scale in terms of airborne SAR to reduce simulation time.

## 3. Simulation Results

### 3.1. SAR Image Simulation of Stationary Target

As a base of SAR image simulation, the SAR signals are simulated in the case of the stationary targets. The compression technique is conducted on the SAR raw signals to obtain fine resolutions. The simulation result is shown to confirm SAR signal processing and to validate whether the simulation is able to obtain settled resolutions.

**Figure 3.**
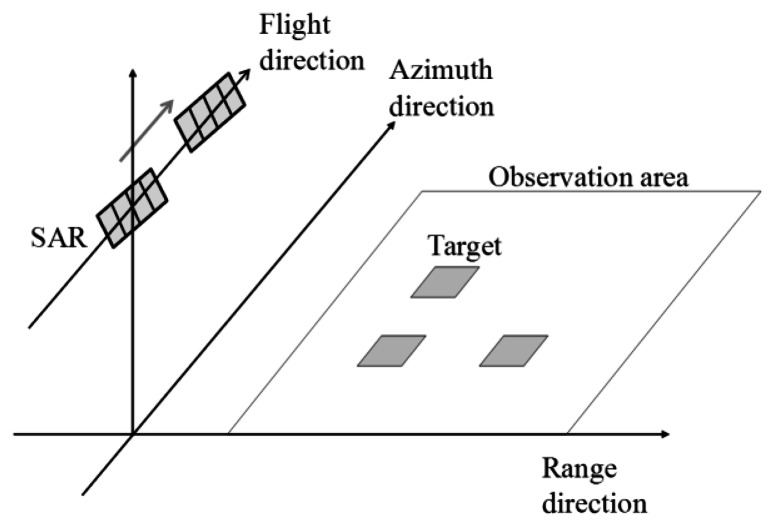
Schematic image of stationary targets.

In the simulation, the stationary targets are flat and square as shown in Figure 3. The scale of the target is 0.5 m. The positions of the targets are located at (1,150 m, 70 m), (1,150 m, 100 m) and (1,200 m, 70 m), that mean (range direction, azimuth direction). The observation areas are 250 m in the range direction and 250 m in the azimuth direction, respectively. We shall ignore microwave backscattering from the surface without the targets to show microwave backscattering of the targets clearly.

**Figure 4.**
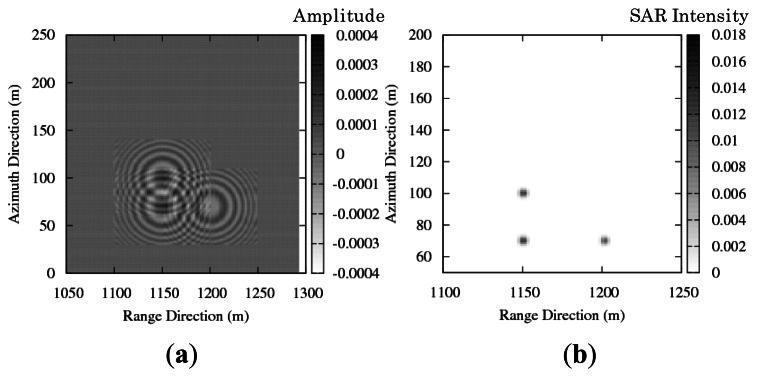
(**a**) Real part of simulated SAR raw signal. (**b**) Intensity of simulated SAR image.

Figure 4(a) shows the SAR raw signals as time series of range and azimuth signals. The simulated signals show chirping and Doppler shift. The SAR images are obtained by compression techniques in the range and the azimuth direction. Figure 4(b) shows the compressed SAR signals by applying the compression technique. The settled resolutions are 4.5 m in the range direction and 3 m in the azimuth direction. The simulation result shows good resolutions in accordance with the settled values. As the result, basic signal processing of the SAR is confirmed by the simulation of stationary targets.

### 3.2. SAR Image Simulation of Moving Target

The targets are treated as moving targets in this section to show azimuthal shift due to the target's velocity. If an observation target is moving, the received signals in the azimuth direction are modulated due to the changes of the relative distances from the target to the antenna during azimuth integration time. It causes displacements in the azimuth signals, and it is called azimuthal shift. The azimuthal shift is described theoretically in case the targets have velocity in the range direction [13,14]. When the velocity of the platform is *V*, the distance from the platform to the moving target is *R*, and if the velocity component of the moving target toward the antenna is *v_r_*, the azimuthal shift is expressed as:

(8) $$Rv_{r}/V$$

The time domain simulation obtains the azimuthal shift because the time series of the received signals include the signal displacements caused by the moving target.

**Figure 5.**
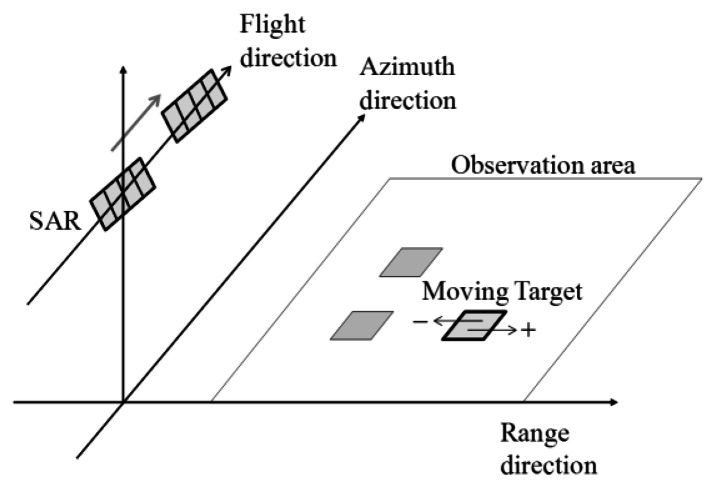
Schematic image of stationary and moving targets.

To examine azimuthal shift in the simulation, the simulation is performed in moving target case. The simulated SAR images are compared with the theoretical azimuthal shift to validate motion effect in simulation results. As illustrated in Figure 5, one target is moving and the other two targets are stationary. The moving target has velocities in positive and negative directions. The simulation parameters are shown in Table 2.

The velocities of the platform and the moving target are *V* = 75 m/s and *v_y_* = ± 0.6 m/s, the incident angle *θ* is 40 degrees. Then, the velocity component toward the platform is *v_r_* = *v_y_*sin*θ*. The distance from the platform to the target is *R* = 1,920.9 m. From Equation (8), the theoretical azimuthal shift is *Rv_r_*/*V* = 9.88 m.

**Figure 6.**
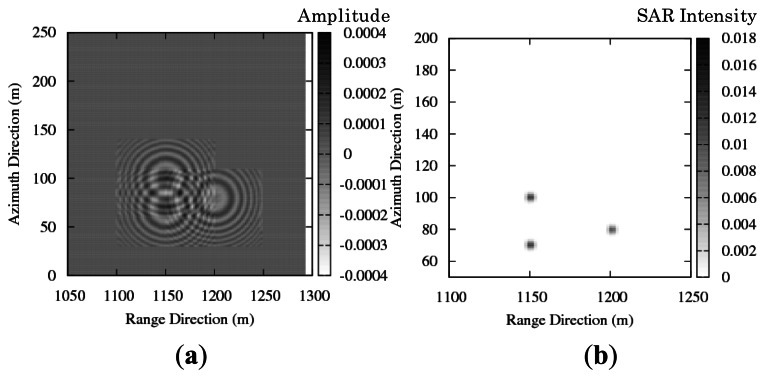
(**a**) Real part of simulated SAR raw signal. (**b**) Intensity of simulated SAR image. (*v*_y_ = + 0.6 m/s).

**Figure 7.**
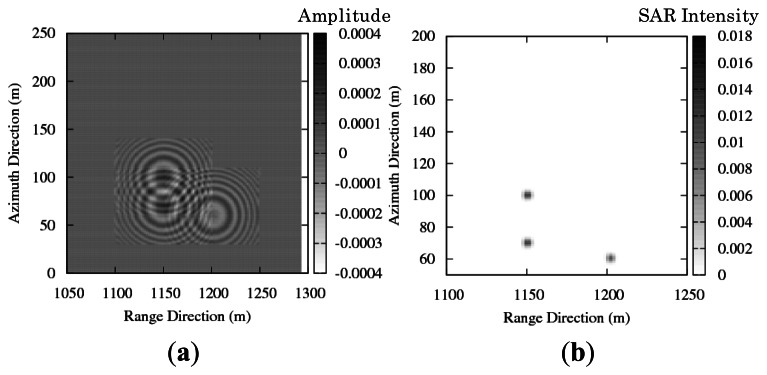
(**a**) Real part of simulated SAR raw signal. (**b**) Intensity of simulated SAR image. (*v*_y_ = −0.6 m/s).

Figures 6(a) and 7(a) show the simulation results of real part of the SAR signals. Figures 6(b) and 7(b) show the simulation results of the SAR image intensity. The azimuthal shift in the simulated images is approx. 10 m. The azimuthal shifts that appeared in the simulation results show consistency with the theoretical values. The direction of azimuthal shift changes relating to radial velocity components; thus, Figures 6 and 7 show the opposite azimuthal shift.

The results show that the time domain simulation is adaptable for the SAR image simulation of the moving targets such as ocean waves. In the next section, we discuss the azimuthal shift from the orbital motions of the ocean waves.

### 3.3. Azimuth Signal Simulation of Moving Ocean Wave

The azimuthal shifts of moving ocean waves cause velocity bunching. It is known that the velocity bunching leads to nonlinear modulation of SAR images, making SAR images of ocean waves difficult to analyze. The velocity components of ocean waves are related to orbital motions; in other words, there are positive and negative velocity components contribute toward the SAR. Therefore they cause various azimuthal shifts, leading to nonlinear modulation due to velocity bunching.

To show the azimuthal shifts of the orbital motions, the azimuth signals are simulated for moving and stationary waves to show the displacements by the orbital motions. The azimuth signals are discussed in the simulation. Thus, the calculation area in the range direction is set at 4.7 m, which is close to that of the azimuth resolution. The incident angle is 35 degrees. The other conditions are the same as those listed in Table 1. The wavelength of the regular wave is 100 m, and the wave height is 1.5 m. The regular wave is traveling to the azimuth direction. The moving wave is expressed as cos(k*x*−ω*t*), and the stationary wave is expressed as cos(k*x*−ω*t*), where *t* and *x* are the values of the time and distance in the azimuth direction.

**Figure 8.**
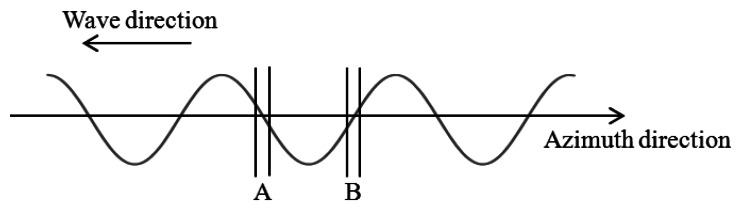
Schematic image of the simulation in the case of regular wave parts.

**Figure 9.**
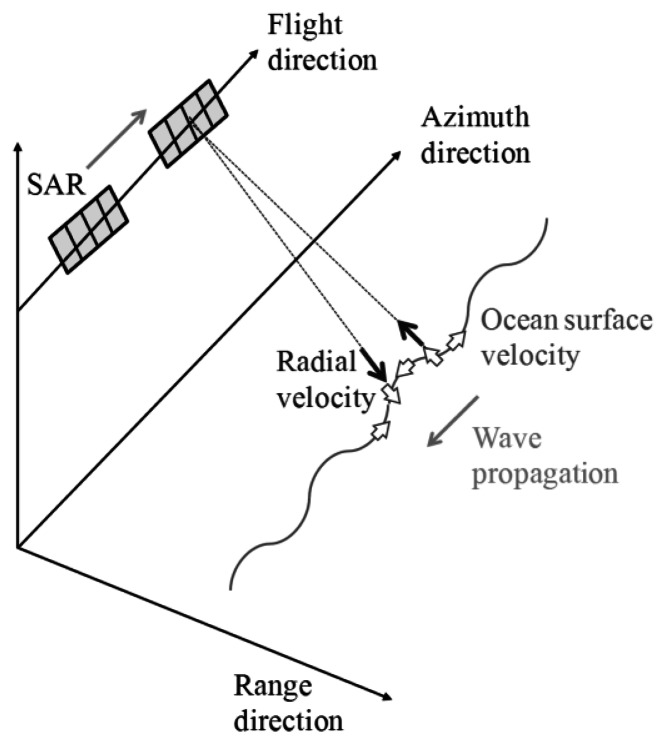
Radial velocity of orbital motions of waves propagating to azimuth direction.

As shown in Figure 8, the small parts (denoted by A and B) of the regular wave are the targets of the simulation. Parts A and B have maximum radial velocities toward the SAR (see Figure 9). These small parts are modeled as elements of long ocean wave. The scale of part A as well as B is at 0.5 m. The simulation is conducted under the condition that part A (or B) is located at 40 m in the azimuth direction from the starting point of the simulation. Microwave backscattering without part A (or B) is neglected to clearly show the azimuthal shifts due to the orbital motions.

**Figure 10.**
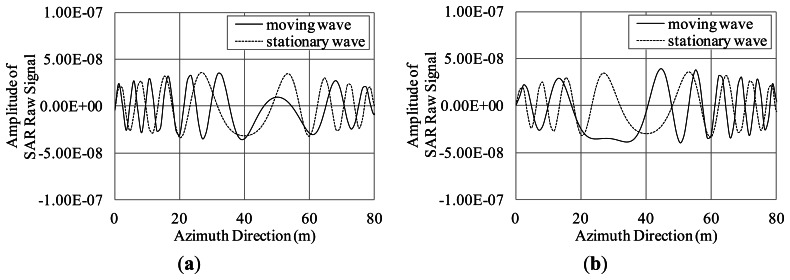
Simulated SAR raw signal of moving wave and stationary wave. (**a**) Part A; (**b**) Part B.

The simulation results shown in Figure 10 are the real parts of the SAR raw signals in the azimuth direction. As evident in the results, the simulated signals of the moving wave are shifted approximately 10 m compared to the stationary wave because of the azimuthal shifts due to the orbital motions. The theoretical azimuthal shifts from the orbital motion in parts A and B are ±11.77 m. The azimuthal shifts of the simulation results are in good agreement with the theoretical values. The simulation results of parts A and B show the opposite azimuthal shifts because the orbital motions of parts A and B have opposite velocities toward the SAR. These shifts may lead bunching at the wave bottom. The results show that the simulation can generate SAR images including azimuthal shifts of ocean waves.

### 3.4. Bragg Scattering

#### 3.4.1. Influence of Bragg Resonant Waves

The primary mechanism of microwave backscattering on the sea surface is Bragg scattering [4]. The key factors of SAR imaging in ocean area are motion induced modulation and backscattering intensity based on the Bragg scattering theory. The Bragg scattering theory states that microwave backscattering emphasizes under the Bragg resonant condition. The Bragg resonant wavelength is given by:

(9) $$\lambda_{e}/(2\sin\theta )$$

**Figure 11.**
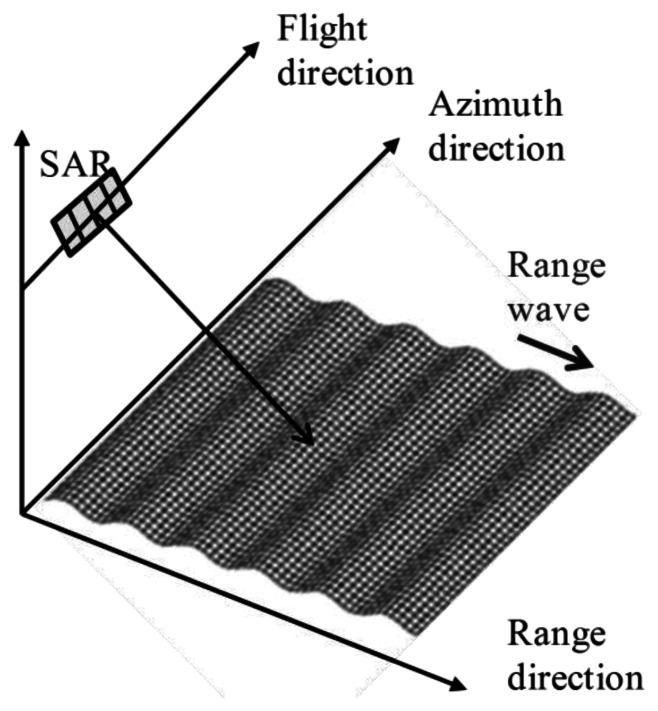
Illustration of range traveling regular wave in Bragg resonant condition.

**Figure 12.**
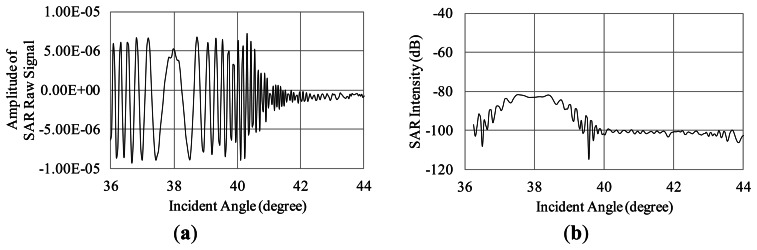
Influence Bragg resonant wave on microwave backscattering (Bragg resonant condition of 38 degrees). (**a**) Real part of SAR raw signal; (**b**) SAR intensity.

**Figure 13.**
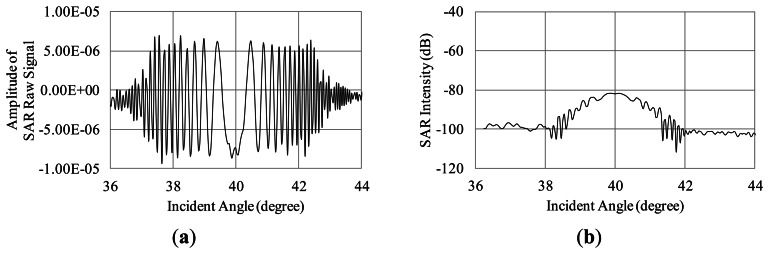
Influence Bragg resonant wave on microwave backscattering (Bragg resonant condition of 40 degrees). (**a**) Real part of SAR raw signal; (**b**) SAR intensity.

**Figure 14.**
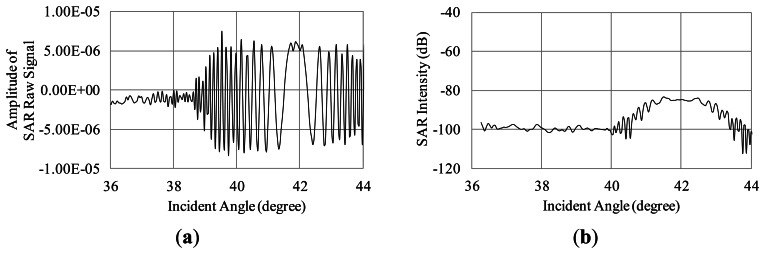
Influence Bragg resonant wave on microwave backscattering (Bragg resonant condition of 42 degrees). (**a**) Real part of SAR raw signal; (**b**) SAR intensity.

To examine the influence of Bragg scattering, the simulation is carried out in the case of the regular waves whose wavelengths are satisfied with the Bragg resonant condition as described in Equation (9). The SAR raw signals in the range direction are simulated for the regular Bragg resonant waves traveling to the range direction (see Figure 11). The simulation conditions are as follows. The incident angles are 36 to 44 degrees. The radar wavelength is 0.235 m. The wavelengths of the regular ocean waves are 0.191, 0.183, 0.176 m, which are the Bragg resonant waves of 38, 40, 42 degrees, respectively. The amplitude is 0.001 m in these three cases. The calculation area in the azimuth direction is 4.7 m. The other conditions of the simulation are the same as Table 1. The SAR raw signals and their compressed signals are shown in Figures 12, 13 and 14.

The results of the compressed signals turn out that the SAR intensity is remarkably increasing at the incident angles of the Bragg resonance. As the results, Bragg scattering is strongly influenced on microwave backscattering from the sea surface. This is caused by emphasis of phases in backscattered signals. The simulation calculates the phases from each computational grid smaller than microwaves to demonstrate the interaction between microwave and ocean surface waves. Thus, the Bragg resonance is occurred in our simulation results. It indicates that the simulation is able to obtain SAR signals based on Bragg resonant scattering.

#### 3.4.2. Numerical Sea Surface

Numerical sea surfaces including the Bragg resonance waves are generated by wind wave spectra. Mitsuyasu and Honda spectrum [16] is used in this study. Mitsuyasu and Honda proposed a high-frequency wave spectrum *S*, which gives frequency and wind speed dependences as the following equation. Bragg resonance is highly occurred in high frequency wave domain so that we use Mitsuyasu and Honda spectrum:

(10) $$S\left(\omega \right)=\alpha_{S}gu_{*}\omega^{-4}$$

where *ω* is the angular frequency of the spectra and u_*_ is the friction velocity of the wind. *α*_s_ is the value relating to u_*_. *g* is the gravitational constant. The parameters used in this simulation are as follows; *U* = 5 m/s, u_*_ = 0.259 m/s, *α*_s_ = 0.0102, where *U* is the wind speed at 0.2 m height [16].

Irregular sea surfaces are expressed as a sum of linear waves, which are divided by wave spectra with a random phase *ε_ij_*. The elevation of the irregular sea surfaces *z* is the following equation (the number of component waves is *N_1_*, the number of directions of component waves is *N_2_*). *x* and *y* are the distance variables, *t* is the time variable:

(11) $$z\left(x,y,t\right)=\sum_{j=1}^{N_{1}}\sum_{i=1}^{N_{2}}\cos\left(k_{i}x\cos\phi_{j}+k_{i}y\text{sin}\phi_{j}-\omega_{i}t+{ɛ}_{ij}\right)\sqrt{2S(\omega_{i})\Delta \omega_{i}}$$

where *ω_i_, k_i_* are the angular frequency and the wave number of component waves. *φ* is the angle between the wave directions and the x-direction, Δ*ω* is the width of the frequency intervals.

For deep water waves, there is the dispersion relationship between the frequencies and the wave numbers:

(12) $$\omega =\sqrt{gk}$$

The details of the numerical wind wave are as follows. The range of wavelength is 0.3 m to 0.15 m, which includes Bragg scattering waves of incident angles of 35 to 45 degrees, and the number of spectral division is 20. The numerical sea surface of wind waves is composed of three directions (±10°). The center direction of the wind wave is toward to the antenna.

**Figure 15.**
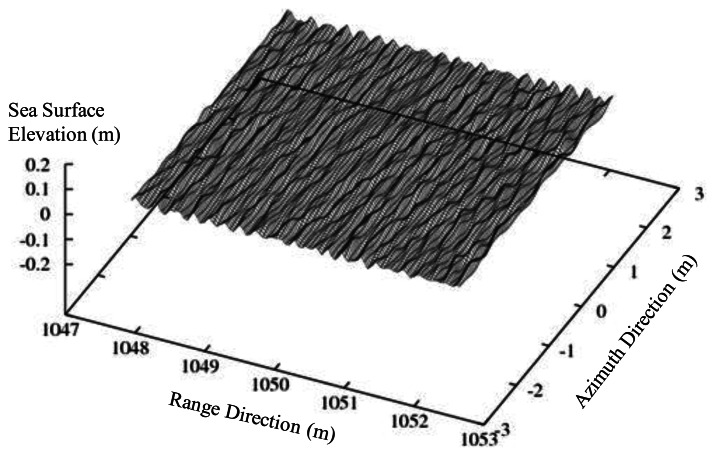
Example of numerical sea surface for irregular wind wave (wind speed 5 m/s).

The examples of the irregular wind waves are shown in Figure 15. Let us note that the numerical sea surface is composed of linear waves, *i.e.*, the numerical sea surface has no breaking. Also, hydrodynamic modulation between small and long wave is not considered, for simplicity.

#### 3.4.3. Comparison with Bragg Scattering Theory

The following equation is the first order of the normalized radar cross section *σ*_0_ of HH polarization [4,17,18]:

(13) $${\sigma_{0}}^{\left(1\right)}\left(\theta \right)=4\pi k^{4}\cos^{4}\theta {\left|\frac{\left({ɛ}_{r}-1\right)}{{\left(\cos\theta +{\left({ɛ}_{r}-\sin^{2}\theta \right)}^{1/2}\right)}^{2}}\right|}^{2}W(2k\sin\theta ,0)$$

where *ε*_r_ is the complex dielectric constant of the ocean, and its value is 73-85*i* in L-band [19]. *W* is the two-dimensional wave number spectra of the ocean surface.

According to Mitsuyasu and Honda [16], the wave number spectra *W*(*k*) can be converted to frequency spectra described in the following equation:

(14) $$W\;(k)=S\;\left(\omega \right)\frac{d\omega }{dk}$$

Radar cross section *σ* represents the ability of scattering microwave energy of the antenna and is defined as:

(15) $$\sigma =4\pi R^{2}\frac{{\left|E_{\text{r}}\right|}^{2}}{{\left|E_{\text{t}}\right|}^{2}}$$

where ***E***_r_ and ***E***_t_ are the received and the transmitted electric fields, respectively. Normalized radar cross section *σ*_0_ is the radar cross section per unit area (pulse irradiation area).

**Figure 16.**
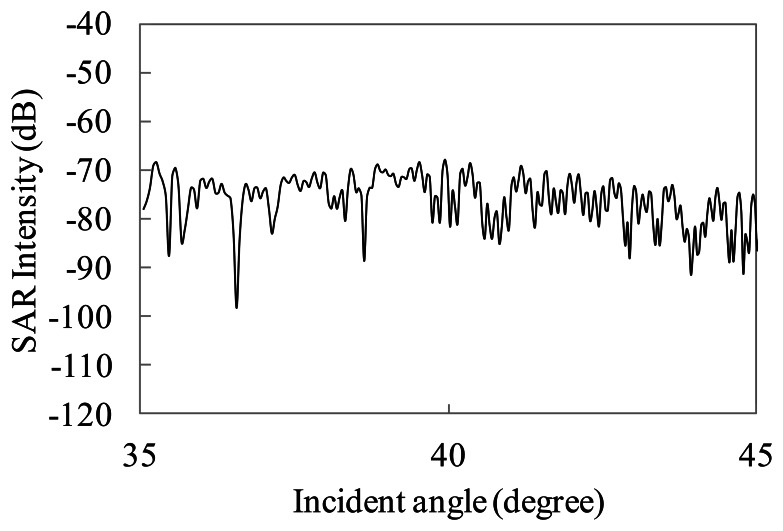
Incident angle dependence of simulated SAR intensity in range direction (wind speed 5 m/s).

The particular microwave backscattering on the sea surface is that the power of the backscattered signals decreases with increase of its incident angles (incident angle dependence). To show scattering features in the simulation, the SAR intensity in the range direction is simulated for wind driven irregular waves. The incident angles of the SAR are 35 to 45 degrees. The wavelengths of the wind waves are the range of 0.3 to 0.15 m, which include Bragg resonance waves. The wind speed is 5 m/s. The simulation conditions listed in Table 1 are used in this section. The incident angle dependences of the simulated SAR intensity in the range direction are shown in Figure 16.

**Figure 17.**
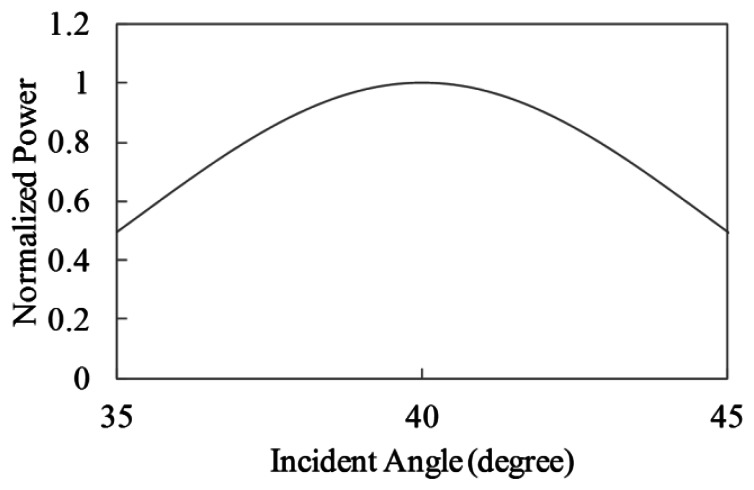
Antenna beam pattern in range direction.

For the sake of comparison with Bragg scattering features, the antenna beam pattern of the range direction shown in Figure 17 is extracted from the simulation results. We remark the incident angle dependence based on Bragg scattering in this paper, then we shall ignore the validation of the speckle in ocean areas.

**Figure 18.**
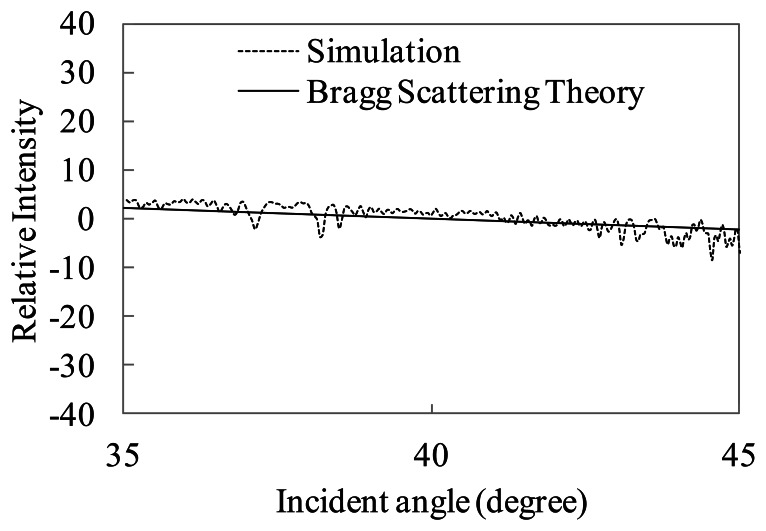
Average value of simulated SAR intensity without antenna beam pattern versus Bragg scattering theory (wind speed 5 m/s).

The incident angle dependence of the simulated SAR intensity without the antenna beam pattern is shown in Figure 18. Figure 18 also presents comparison with normalized radar cross section calculated by Bragg scattering theory described in Equation (13). The wind speed is also 5 m/s. The dotted line shows the simulated SAR intensity. The simulation result is the average of 10 range lines with varying the irregular sea surfaces. The solid line shows the theoretical values of Bragg scattering. The figure shows the relative intensity, which is the deviation of mean value between 35 to 45 degrees, to compare the simulation with the theoretical incident angle dependence.

Based on Figure 18, the intensities in the incident angle dependence are gradually decreasing. The decreasing rate shows good tendency with the theoretical line. The simulation result of incident angle dependence is in good agreement with the Bragg scattering. The simulation by physical optics considers phases of backscattered microwaves. Therefore, the simulation is able to calculate microwave backscattering associated with the Bragg scattering.

These simulation results turn out that the simulation is able to obtain microwave backscattering as SAR signals with regard to backscattering intensity and motion induced modulations. As a next step, SAR imaging mechanism for ocean surface will be discussed using this simulation.

## 4. Conclusions

The fundamental simulation technique has been developed to obtain SAR images for moving ocean surfaces. The simulation has been designed in time domain to consider modulation caused by the orbital motions of ocean waves. In addition, the simulator aims to be based on Bragg scattering. These are the key factors of SAR image in oceanic scenes.

The time series of the SAR raw signals are first simulated for stationary targets to confirm basic SAR signal processing. In the simulation results of moving targets, the backscattered signals from the moving target are azimuthally modulated by the radial velocity component of the moving target. The displacements of the azimuthal shifts in the simulation are consistent with the theoretical values. In addition, the azimuth signals are simulated for regular waves to show the azimuthal shifts caused by the orbital motions. Furthermore, SAR intensities in range direction are simulated for wind waves to prove that the simulation is based on the Bragg scattering. As for the results, incident angle dependence shows good tendency with the result obtained using the Bragg scattering theory. The simulation results show that the simulation is in good agreement with the theories of azimuthal shift and Bragg scattering. Therefore, the time domain simulation technique is used successfully to obtain numerical SAR images with regard to motion effects and Bragg scattering. It is expected that the simulation will help in understanding the imaging mechanism of SAR images in oceanic scenes. Furthermore, analytical algorithms retracing sea surface data from the SAR images will be evaluated by using the simulated SAR images.

## Figures and Tables

**Table 1.** Simulation conditions.

Parameter	Value
Radar frequency	1.275 GHz
Radar wavelength	0.235 m
Polarization	HH
Look angle	40 deg
Range resolution	4.5 m
Azimuth resolution	3 m
Pulse duration	0.2 μs
Pulse reputation frequency	63.8 Hz
Chirp rate	250 × 10^12^ Hz/s
Chirp band width	50 MHz
Sampling frequency	255.3 MHz
Altitude of platform	1,500 m
Velocity of platform	75 m/s
Calculation area	Range : 100 m
(Pulse irradiation area)	Azimuth : 80 m
Scale of computational grid	0.047 m
Range beam width	10.0 deg
Range antenna length	1.2 m
Azimuth beam width	2.0 deg
Azimuth antenna length	6.0 m

**Table 2.** Simulation parameters of moving target case.

Parameter	Value
Platform velocity	75 m/s
Distance from target	1920.94 m
Target velocity	± 0.6 m/s
Incident angle	40 degrees
Radial velocity of target	0.39 m/s
Theoretical azimuth shift	9.88 m
